# Color Trails Test: A New Set of Data on Cognitive Flexibility and Processing Speed in Schizophrenia

**DOI:** 10.3389/fpsyt.2020.00521

**Published:** 2020-06-09

**Authors:** Ernest Tyburski, Ewa Karabanowicz, Monika Mak, Zofia Lebiecka, Agnieszka Samochowiec, Justyna Pełka-Wysiecka, Leszek Sagan, Jerzy Samochowiec

**Affiliations:** ^1^Institute of Psychology, SWPS University of Social Sciences and Humanities, Poznań, Poland; ^2^Institute of Psychology, University of Szczecin, Szczecin, Poland; ^3^Independent Clinical Psychology Unit, Pomeranian Medical University, Szczecin, Poland; ^4^Department of Psychiatry, Pomeranian Medical University, Szczecin, Poland; ^5^Department of Neurosurgery, Pomeranian Medical University, Szczecin, Poland

**Keywords:** cognitive flexibility, processing speed, executive functions, cognitive functions, schizophrenia

## Abstract

**Background:**

Although schizophrenia patients have been reported to manifest deficits in cognitive flexibility and lower processing speed (measured with i.a., the Color Trails Test, CTT), there still remain a few matters that require further investigation. We have therefore formulated three research aims: 1) to examine the factor structure of CTT in schizophrenia patients and healthy controls, 2) to compare different CTT performance measures in the two groups, 3) to investigate the relationship between these measures and selected psychopathological symptoms in the patient group.

**Methods:**

Sixty-seven patients with paranoid schizophrenia and 67 healthy controls, matched for gender, age, number of years of education, and overall cognitive functioning underwent assessment of cognitive flexibility and processing speed with the CTT.

**Results:**

Factor analysis of CTT variables based on the principal component method revealed a four-factor solution in both groups. Compared with healthy controls, the patients performed poorer on CTT 1 time, CTT 2 time, 2-1 difference, prompts in CTT 2, and had higher regression factor scores for Factor 1 (reflecting the slower speed of perceptual tracking). Furthermore, significant links were found between some CTT measures, and negative and disorganization symptoms.

**Conclusions:**

Schizophrenia patients exhibit problems with speed of perceptual tracking and executive processes dependent on processing speed. Our results may be useful for the development of neuropsychological diagnostic methods for schizophrenia patients. It seems that, compared to other CTT indices, CTT 1 time, CTT 2 time, and 2-1 difference are more appropriate measures of cognitive performance in schizophrenia patients.

## Introduction

Cognitive flexibility is defined as the ability to flexibly transition between at least two processes or tasks [i.e., alternating attention between two goals (1)]. Processing speed refers to the number of correct responses an individual is able to generate during a task within a given amount of time or the speed with which different cognitive operations can be performed [i.e., motor speed and visual scanning speed (2, 3)]. As a consequence of structural and functional abnormalities in the brain, both processes may be severely disturbed in schizophrenia (4, 5).

These processes can be measured with two corresponding neuropsychological tests—the Color Trails Test [CTT, (6)] and the Trail Making Test [TMT, (7)]. Introduced in the 1990s, the CTT was primarily created to meet the demand for a culture-neutral equivalent to the TMT. Even though their scores are comparable in most tested populations, CTT’s lack of numbers and letters and use of colors for responses seems to place people from cultures which don’t use the Latin alphabet, illiterate persons, or those experiencing language difficulties at an advantage (6). Initially, performance times in both parts of these tests were interpreted separately, with CTT 1 and TMT A considered to measure processing speed, while CTT 2 and TMT B to assess cognitive flexibility (8–10).

Although the CTT and TMT are reported to measure various neuropsychological domains, data concerning their factorial validity when applied in clinical samples is still scarce. Internal structures of CTT investigated in the normative vs. traumatic brain injury patient samples proved somewhat divergent, although both yielded four-factor solutions, reflecting the speed of perceptual tracking and sequencing (6). To our knowledge, a factorial analysis of CTT has not been conducted yet in schizophrenia patients.

To date, the CTT was used less often than the TMT to assess response time-dependent mental processes in schizophrenia patients (8, 11). A PubMed search results indicate differences between patients and healthy controls in terms of response times in CTT 1 and/or CTT 2 (12–18), though some studies seem to yield conflicting results (19, 20). Apart from these two indices, other researchers have proposed three derived scores to provide information on cognitive flexibility somewhat independent of motor and visual scanning speed [e.g., 2-1 difference, 2/1 ratio, and (2-1)/1 proportion]. There is, however, little research on their use in schizophrenia (13, 16, 21, 22).

Assessment of cognitive flexibility based on CTT or TMT performance times as a single outcome measure is bound to be limited. In turn, a more qualitative approach can offer much more useful clinical information (1), as suggested in several studies, where CTT and TMT error analysis in clinical samples, including schizophrenia, resulted in increased diagnostic specificity of cognitive testing (13, 23–27).

Although deficient cognitive flexibility and processing speed have been reported in schizophrenia, their association with psychopathology [especially different symptom dimensions, not only negative symptoms, see (28)] remains unclear. What is more, there are few findings reporting links between patients’ performance on the CTT and selected clinical variables (19, 29). In contrast, many studies suggest positive correlations between performance time in TMT part B, and negative and disorganization symptoms (30).

Considering all these findings and inconsistencies, we have formulated the main objective of this study, i.e., to examine the factor structure of CTT in schizophrenia patients and healthy controls. Our secondary aims were to compare different CTT performance measures in the two groups and explore their links with selected psychopathological symptoms in the patient group.

## Methods

### Participants

Sixty-seven inpatients of three psychiatric wards were diagnosed with paranoid schizophrenia according to ICD-10 diagnostic criteria [World Health Organization (WHO), (31)] by three licensed psychiatrists. Inclusion criteria were 20–35 years of age, comprehension of test procedure, and stable clinical status. Exclusion criteria were comorbid mental or neurological disorders, craniocerebral injuries, dementia, severe somatic diseases (e.g., cancer), and addiction to alcohol or other substances. All patients gave written informed consent to participate in the study. The study protocol was approved by the local bioethics committee.

Sixty-seven persons without mental or neurological disorders, matched for gender, age, and number of years of education were recruited through information spread by students of the local universities. Inclusion and exclusion criteria for healthy controls were the same as those for patients (except for clinical parameters regarding the diagnosis of schizophrenia).

### Neuropsychological Assessment

Polish Version of CTT by Łojek and Stańczak (6, 13) was used to measure cognitive flexibility and processing speed. The CTT is deemed a culture-free version of the TMT and consists of two parts: (a) CTT 1 requires participants to connect a series of 25 numbered circles that are randomly printed on a sheet of paper and (b) in CTT 2 they are to connect numbered circles from 1 to 25 alternating between two colors—pink and yellow. In this study, we used time-related indices: CTT 1 time, CTT 2 time, 2-1 difference proposed by Chan (19), 2/1 ratio adopted from Lamberty (32), (2-1)/1 proportion proposed by D’Elia (6), and error-related ones: number sequence errors in CTT 1 and/or CTT 2 (when participants connected circles in a wrong order, e.g., 1-3 or 2-4), color sequence errors in CTT 2 (when participants connected circles in a wrong color sequence e.g., 1 pink-2 pink), near-misses in both parts of CTT (when participants started connecting circles in a wrong manner but did correct their work), and prompts in both parts of this test (when participants failed to connect circles for 10 sec, the researcher indicated the next move), as suggested by D’Elia et al. (6).

### Clinical Assessment

The Positive and Negative Syndrome Scale [PANSS, (33)] was used to measure psychopathological symptom severity in schizophrenia patients. Following Emsley et al. (34), we distinguished five psychopathological dimensions: negative, positive, disorganized, excited, anxiety and depression. In addition, Mini-Mental State Examination [MMSE, (35)] was used to screen for global cognitive function.

### Statistical Analysis

Statistical analysis of the data was conducted using the IBM SPSS 24 Statistical package. To investigate the factor structure, 12 CTT scores (see **Table 2**) were submitted to principal components analysis with VARIMAX rotation. Factors with eigenvalues > 1 (the Kaiser-Guttman criterion) were retained and factor loadings of.40 or greater were considered significant. Indices and factors (regression factor scores) of CTT were used as dependent variables. To check for differences between the groups, we used Student’s *t-*test. Cohen’s *d* was used to determine the magnitude of effect size measures (36). Holm’s *p*-value correction for multiple comparisons was used (37). To assess the strength of the identified correlations, The Pearson *r* correlation coefficient was used. In the case of significant correlations, single stepwise linear regression procedures were conducted, in the schizophrenia group.

## Results

### Participant Characteristics

Schizophrenia patient group included 31 women and 36 men, while the control group comprised 27 women and 40 men. The groups did not differ in terms of gender, age, years of education or global cognitive function (MMSE score, see **Table 1**).

**Table 1:** Demographic, clinical, psychological characteristics, and performance in the Color Trail Test (CTT) in schizophrenia patients and healthy controls.

Variable/measure	Schizophrenia patients		Healthy controls		*t*	*d*
	*M*	*SD*	*M*	*SD*		
**Demographic and psychological characteristics**						
Age	28.73	4.27	28.67	4.56	0.08	–
Years of education	14.63	2.80	15.21	2.61	-1.25	–
General cognitive functioning	28.37	1.64	28.72	1.28	-1.35	–
**Clinical characteristics**						
Duration of illness	5.08	4.55	min-max: 1 - 17			
Psychopathological symptoms in PANSS						
Positive symptoms	4.60	5.55	min-max: 0 - 25			
Negative symptoms	7.64	6.27	min-max: 0 - 29			
Disorganization symptoms	4.19	4.03	min-max: 0 - 14			
Excited symptoms	1.60	2.75	min-max: 0 - 15			
Anxiety and depression symptoms	3.66	2.84	min-max: 0 - 15			
**Time-based indices**						
CTT 1 time	54.10	21.90	36.03	14.47	5.64***	0.98
CTT 2 time	112.64	46.03	67.16	23.30	7.22***	1.26
2-1 difference	58.54	32.05	31.13	18.42	6.09***	1.06
2/1 ratio	2.17	0.60	1.96	0.60	1.97	–
(2-1)/1 proportion	1.17	0.60	0.96	0.60	1.97	–
**Error-based indices**						
CTT 1 number sequence errors	0.07	0.26	0.15	0.40	-1.28	–
CTT 1 near-misses	0.21	0.59	0.24	0.50	-0.32	–
CTT 1 prompts	0.19	0.70	0.06	0.24	1.48	–
CTT 2 number sequence errors	0.07	0.32	0.06	0.30	0.28	–
CTT 2 color sequence errors	0.33	0.59	0.12	0.37	2.46	–
CTT 2 near-misses	0.16	0.41	0.19	0.43	-0.41	–
CTT 2 prompts	0.69	1.38	0.10	0.35	3.34**	0.58
**Indices based on factor structure**						
Regression factor scores for Factor 1	0.44	1.19	-0.44	0.47	5.60***	0.97
Regression factor scores for Factor 2	0.15	1.00	-0.15	0.98	1.78	–
Regression factor scores for Factor 3	-0.08	1.00	0.08	1.00	-0.96	–
Regression factor scores for Factor 4	0.21	1.08	-0.21	0.87	2.52	–

PANSS, Positive and Negative Syndrome Scale. Regression factor scores means with 95% confidence intervals for Factor 1, Factor 2, Factor 3, and Factor 4.

**p < 0.01. ***p < 0.001.

### Factor Structure of CTT

We conducted exploratory factor analysis separately for the entire sample, schizophrenia patients and healthy controls. The four-factor solution explained 72.39% of the total variance in the entire sample, 73.26% in schizophrenia patients, and 71.70% in healthy controls. The distribution of the explained variance of the 4 factors in the two subgroups remained approximately the same as for the entire sample. Factor loading estimates for the whole sample and the two subgroups are shown in **Table 2** for each of the four factors, respectively. Factor structures were somewhat divergent in the two subgroups, although both yielded four-factor solutions. Factor 1 reflected speed of perceptual tracking, Factor 2 - cognitive flexibility independent of processing speed, Factor 3 - inattention and impulsivity, and Factor 4 - simple inattention.

**Table 2:** Factor loadings of Color Trail Test (CTT) scores for the entire sample, schizophrenia patients and healthy controls.

Measure	Component			
	Factor 1	Factor 2	Factor 3	Factor 4
**Entire sample**				
CTT 2 time	0.865			
CTT 1 time	0.828			
CTT 2 prompts	0.813			
2-1 difference	0.681	0.671		
CTT 1 prompts	0.660			
2/1 ratio		0.995		
(2-1)/1 proportion		0.995		
CTT 1 near-misses			0.838	
CTT 2 near-misses			0.772	
CTT 2 color sequence errors				0.706
CTT 2 number sequence errors				0.597
CTT 1 number sequence errors			0.443	-0.475
Variance (%) explained by each factor	25.60%	22.15%	12.95%	11.69%
Cumulative explained variance %	25.60%	47.75%	60.70%	72.39%
**Schizophrenia patients**				
CTT 2 time	0.938			
CTT 1 time	0.852	-0.407		
2-1 difference	0.765	0.607		
CTT 2 prompts	0.751			
(2-1)/1 proportion		0.991		
2/1 ratio		0.991		
CTT 1 near-misses			0.864	
CTT 2 near-misses			0.710	
CTT 2 color sequence errors			0.509	
CTT 1 number sequence errors				0.698
CTT 1 prompts	0.525			0.617
CTT 2 number sequence errors				-0.405
Variance (%) explained by each factor	26.23%	21.91%	13.45%	11.67%
Cumulative explained variance %	26.23%	48.14%	61.59%	73.26%
**Healthy controls**				
CTT 1 time	0.823			
CTT 2 time	0.645	0.688		
CTT 1 prompts	0.639			
CTT 2 prompts	0.439			
2-1 difference		0.972		
(2-1)/1 proportion		0.942		
2/1 ratio		0.942		
CTT 1 near-misses			0.780	
CTT 2 near-misses			0.744	
CTT 1 number sequence errors			0.662	
CTT 2 number sequence errors				0.833
CTT 2 color sequence errors				0.831
Variance (%) explained by each factor	16.15%	27.24%	15.00%	13.31%
Cumulative explained variance %	16.15%	43.39%	58.39%	71.70%

### Performance on Different CTT Measures

#### Response Time Indices

As shown in **Table 1**, compared to healthy controls, schizophrenia patients scored poorer on response time-based indices: CTT 1 time (*p* < 0.001), CTT 2 time (*p* < 0.001), and 2-1 difference (*p* < 0.001). The effect sizes (*d*) of the analysed processes were found to be 0.98–1.26, thus suggesting large effect sizes.

#### Error-Based Indices

Compared to healthy controls, schizophrenia patients required significantly more prompts (*p* < 0.01) only in CTT 2. The effect size (*d*) of the analysed errors was found to be 0.58, indicating medium effect sizes.

#### Factor Indices

Regression factor scores for Factor 1 (*p* < 0.001) were significantly higher in the patients than the controls. The effect size (*d*) of the analysed scoring was found to be 0.97, indicating large effect sizes.

### CTT Indices and Psychopathological Symptoms

Negative symptoms were significant predictors of CTT 2 time (β = 0.28; *t* = 2.36; *p* = 0.021) and 2-1 difference (β = 0.27; *t* = 2.26; *p* = 0.027), predicting about 7 and 6% of variance, respectively. Disorganization symptoms were significant predictors of CTT 2 time (β = 0.38; *t* = 3.33; *p* = 0.001), 2-1 difference (β = 0.39; *t* = 3.39; *p* = 0.001), and regression factor scores for Factor 1 (β = 0.24; *t* = 2.01; *p* = 0.049), predicting 13, 14, and 4% of variance, respectively. Excited symptoms were a significant predictor of near-misses (β = 0.30; *t* = 2.57; *p* = 0.012), predicting 8% of variance. We identified no other significant predictors of any CTT indices.

## Discussion

Our primary purpose was to examine the factor structure of CTT in the entire sample and the two subgroups. Factor structures in the entire sample, schizophrenia patients, and healthy controls were similar (Factor 1 = speed of perceptual tracking, Factor 2 = cognitive flexibility independent of processing speed, Factor 3 = inattention and impulsivity, and Factor 4 = simple inattention), suggesting that the error and prompt variables might tap factors dissociable from those based on the time variables. In addition, these qualitative variables may offer an alternative take on the speed vs. accuracy trade-off of CTT performance. Respondents may attempt to increase their speed at the expense of committing errors, or reduce the number of errors at the expense of elevated time scores. Our results are thus similar to those reported in the traumatic brain injury and normative samples (6).

Our second aim was to compare CTT performance measures in schizophrenia patients and healthy controls. In this study was used CTT, as performance on CTT 2 is considered to be more perceptually demanding than completion of TMT B [49 vs. 25 circles, respectively; (38)]. Furthermore, the CTT allows the use of more qualitative scoring than the TMT, e.g., number or color sequence errors, near-misses, and prompts (6). Patients performed poorer on three time-based indices (CTT 1 time, CTT 2 time, and 2-1 difference), which is partly consistent with findings reported by other researchers (12, 14, 15, 17, 18). Despite the between-group differences regarding the 2/1 ratio and the (2-1)/1 proportion, after Holm’s *p*-value correction, the differences were no longer statistically significant, which is consistent with other reports (13, 16). Such results are further confirmed by higher regression factor scores for Factor 1 indicates the slower speed of perceptual tracking in schizophrenia patients. Our results are in-line with the data reported by other researchers, especially Dickinson et al. (39, 40). Furthermore, patients’ poorer performance could result from their impaired working memory or visual-spatial functions, described elsewhere (11). Their executive deficits may stem from improper communication between different cortical and subcortical structures of the brain (41).

Our third purpose was to study the links between all CTT indices and selected psychopathological symptoms. Although Chan et al. (19) report no correlations with psychopathological symptoms, we found associations between negative and disorganization symptoms and lower processing speed as well as impaired cognitive flexibility. Our findings are thus similar to those reported in a meta-analysis by Dibben et al. (30), although those authors used the TMT. The observed links between excited symptoms and near-misses could reflect poor impulse control.

We should take account of the study limitations. Firstly, the sample included patients with paranoid schizophrenia, which may hinder generalization to the entire schizophrenia population. Secondly, we did not include analyses of other neurocognitive tests or make a discriminant or convergent validity analysis. Thirdly, the measures-to-person ratio in the CTT in both groups was low but acceptable (42).

To conclude, the major value of this study is that it provides a new set of clinical and non-clinical data which may be of use for neuropsychologists, clinicians, and psychiatrists to determine more precisely the extent to which CTT 1 and CTT 2 scores, and derived indices reflect patient impaired performance. It seems that using CTT 1 time, CTT 2 time, and 2-1 difference are more appropriate measures of cognitive performance dependent on CTT in schizophrenia. The main finding of this study is also that schizophrenia is primarily characterized by problems in the speed of perceptual tracking and executive processes dependent on processing speed.

## Data Availability Statement

The datasets generated for this study are available on request to the corresponding author.

## Ethics Statement

This study was carried out in accordance with the recommendations of Bioethical Commission of the Institute of Psychology of University of Szczecin with written informed consent from all subjects. All subjects gave written informed consent in accordance with the Declaration of Helsinki. The protocol was approved by the Bioethical Commission of the Institute of Psychology of University of Szczecin.

## Author Contributions

All authors contributed to and have approved the final manuscript. ET was the principal coordinator of the grant, was involved in the study design, and took part in patients recruitment, managed literature searches and analyses, performed the statistical analysis, wrote the first draft of the manuscript. EK was involved in the conceptualization of the project and took part in patients recruitment. MM was involved in the study design and corrected the manuscript. ZL corrected the manuscript. AS corrected the manuscript. JP-W was involved in the study design and corrected the manuscript. LS corrected the manuscript. JS was involved in the study design and corrected the manuscript.

## Funding

This work was supported by the Faculty of Humanities at the University of Szczecin (504-3000-240-940/2015 and 504-3000-240-940/2016) and the Pomeranian Medical University in Szczecin (FSN-337-06/2016 and FSN-246-05/2017). The project was also financed by the Polish Minister of Science and Higher Education’s program named “Regional Initiative of Excellence” in 2019–2022 (002/RID/2018/19).

## Conflict of Interest

The authors declare that the research was conducted in the absence of any commercial or financial relationships that could be construed as a potential conflict of interest.
